# Supplementary material for: Genomic Landscape and Mutational Spectrum of ADAMTS Family Genes in Mendelian Disorders Based on Gene Evidence Review for Variant Interpretation
Source: Biomolecules. 2020 Mar 13;10(3):449. doi: 10.3390/biom10030449 (PMC7175297; doi:10.3390/biom10030449)
Supplement: Supplementary file 1 [file biomolecules-10-00449-s001.pdf]

Supplementary Table 1: Reassessment of variants in ADAMTS family genes with strong evidence for Mendelian disorders previously suggested for association but disqualified for pathogenicity using ACMG variant interpretation guideline.

Gene	Transcript	Nucleotide change	Amino acid change	Conservation*				Population database			Prediction algorithms		ACMG guideline	Reasons for disqualification
				Mm	Gg	Xt	Dr	gnomAD_all	gnomAD_maxPop	dbSNP	SIFT	PP2		
ADAMTS2	NM_014244.4	c.3328C>G	p.P1110A	P	L	_	_	none		none	Tol (0.29)	Ben (0.001)	Variant of unknown significance	Incorrect disease association, Weak clinical data
ADAMTS3	NM_014243.2	c.2786G>A	p.R929H	R	R	R	R	0.0008%	SAS:0.0033%	rs780251054	Del (0.01)	Dam (0.971)	Variant of unknown significance	Assocation study, Single case with de novo variant
ADAMTS9	NM_182920.1	c.4543C>T	p.R1515*	NA	NA	NA	NA	none		none	NA	NA	Variant of unknown significance	Incorrect disease association, Weak clinical data
ADAMTS10	NM_030957.3	c.1549G>A	p.E517K	E	E	E	E	none		none	Tol (0.09)	Dam (0.980)	Variant of unknown significance	Incorrect disease association, Weak clinical data
ADAMTS10	NM_030957.3	c.3303_3311delTGGCCACTA	p.H1101_H1103delins36	NA	NA	NA	NA	none		none	NA	NA	Variant of unknown significance	In-frame variant at the end of gene
ADAMTS17	NM_139057.3	c.2878A>G	p.R960G	R	R	R	R	none		none	Del (0)	Dam (0.516)	Variant of unknown significance	Incorrect disease association, Weak clinical data
ADAMTS18	NM_199355.3	c.113G>A	p.C38Y	C	_	C	_	0.0034%	EAS:0.042%	rs540472609	Tol (0.24)	Ben (0.077)	Variant of unknown significance	Single case with no additional pathogenic mutation
ADAMTS18	NM_199355.3	c.303T>A	p.F101L	V	F	F	F	none		none	Tol (1)	Ben (0.002)	Variant of unknown significance	Incorrect disease association, Weak clinical data
ADAMTS18	NM_199355.3	c.1985C>G	p.P662R	P	P	P	P	0.0170%	EAS:0.18%	rs747420099	Del (0)	Ben (0.168)	Variant of unknown significance	Single case with no additional pathogenic mutation
ADAMTS18	NM_199355.3	c.3300_3303dupGAAA	p.P1102Efs*73	NA	NA	NA	NA	none		none	NA	NA	Variant of unknown significance	Incorrect disease association, Weak clinical data

ADAMTS19	NM_133638.4	c.1653delT	p.P552Rfs*7	NA	NA	NA	NA	none		none	NA	NA	Variant of unknown significance	Incorrect disease association, Weak clinical data
ADAMTS19	NM_133638.4	c.1984C>T	p.R662*	NA	NA	NA	NA	0.0008%	NFE:0.0018%	rs772148624	NA	NA	Variant of unknown significance	Incorrect disease association, Weak clinical data

Abbreviations: NA, not available; Tol, tolerated; Del, deleterious; Ben, benign; Dam, damaging

* Codes in conservation columns represent the corresponding amino acids in four species at the positions of mutation according to standard amino acid abbreviations by IUPAC-IUB Joint Commission on Biochemical Nomenclature.

Supplementary Table 2: List of 202 pathogenic mutations in *ADAMTS13* (NM_139025.4) with strong evidence for hereditary thrombotic thrombocytopenic purpura or Upshaw-Schulman syndrome.

Nucleotide change	Amino acid change	Type of mutation
c.1039T>A	p.C347S	Missense
c.1045C>T	p.R349C	Missense
c.1058C>T	p.P353L	Missense
c.1154G>A	p.G385E	Missense
c.1170G>C	p.W390C	Missense
c.1192C>T	p.R398C	Missense
c.1193G>A	p.R398H	Missense
c.1198T>C	p.C400R	Missense
c.1201G>A	p.G401R	Missense
c.1225C>T	p.R409W	Missense
c.1297T>C	p.C433R	Missense
c.1297T>G	p.C433G	Missense
c.1308G>C	p.Q436H	Missense
c.1313G>A	p.C438Y	Missense
c.1368G>T	p.Q456H	Missense
c.1370C>T	p.P457L	Missense
c.1492C>T	p.R498C	Missense
c.1520G>A	p.R507Q	Missense
c.1523G>A	p.C508Y	Missense
c.1574G>A	p.G525D	Missense
c.1582A>G	p.R528G	Missense
c.1624T>G	p.W542G	Missense
c.1648G>A	p.G550R	Missense
c.1709A>G	p.Y570C	Missense
c.1787C>T	p.A596V	Missense
c.1816G>C	p.A606P	Missense
c.1892C>T	p.A631V	Missense
c.1921G>A	p.E641K	Missense
c.1973A>G	p.Y658C	Missense
c.1976G>A	p.R659K	Missense
c.2012C>T	p.P671L	Missense
c.2017A>T	p.I673F	Missense
c.2068G>A	p.A690T	Missense
c.2074C>T	p.R692C	Missense
c.2104G>C	p.G702R	Missense
c.2167C>A	p.Q723K	Missense
c.2195C>T	p.A732V	Missense
c.2209T>C	p.C737R	Missense
c.2218G>A	p.E740K	Missense
c.2260T>C	p.C754R	Missense

c.2272T>C	p.C758R	Missense
c.2281G>A	p.G761S	Missense
c.2293C>T	p.R765W	Missense
c.237C>G	p.I79M	Missense
c.2410T>C	p.C804R	Missense
c.262G>A	p.V88M	Missense
c.262G>C	p.V88L	Missense
c.2701G>T	p.A901S	Missense
c.2708C>T	p.S903L	Missense
c.2723G>A	p.C908Y	Missense
c.2723G>C	p.C908S	Missense
c.2724C>G	p.C908W	Missense
c.2725G>A	p.G909R	Missense
c.2746C>T	p.R916C	Missense
c.2836T>C	p.C946R	Missense
c.283G>C	p.A95P	Missense
c.2851T>G	p.C951G	Missense
c.286C>G	p.H96D	Missense
c.2914C>T	p.R972W	Missense
c.2930G>T	p.C977F	Missense
c.304C>T	p.R102C	Missense
c.305G>A	p.R102H	Missense
c.3070T>C	p.C1024R	Missense
c.3070T>G	p.C1024G	Missense
c.3092G>A	p.G1031D	Missense
c.3178C>T	p.R1060W	Missense
c.3251G>A	p.C1084Y	Missense
c.3283C>T	p.R1095W	Missense
c.3284G>A	p.R1095Q	Missense
c.3367C>T	p.R1123C	Missense
c.3368G>A	p.R1123H	Missense
c.3541G>A	p.G1181R	Missense
c.356C>T	p.S119F	Missense
c.3638G>A	p.C1213Y	Missense
c.3650T>C	p.I1217T	Missense
c.3655C>T	p.R1219W	Missense
c.3656G>A	p.R1219Q	Missense
c.3716G>T	p.G1239V	Missense
c.4006C>T	p.R1336W	Missense
c.4085A>T	p.D1362V	Missense
c.4156G>C	p.A1386P	Missense
c.4184T>C	p.L1395P	Missense
c.4205G>C	p.R1402P	Missense
c.427A>T	p.I143F	Missense
c.428T>C	p.I143T	Missense

c.448T>C	p.S150P	Missense
c.518A>G	p.D173G	Missense
c.530A>G	p.Y177C	Missense
c.533T>C	p.I178T	Missense
c.559G>C	p.D187H	Missense
c.577C>T	p.R193W	Missense
c.578G>A	p.R193Q	Missense
c.581G>T	p.G194V	Missense
c.587C>T	p.T196I	Missense
c.607T>C	p.S203P	Missense
c.649G>C	p.D217H	Missense
c.679G>A	p.G227R	Missense
c.695T>A	p.L232Q	Missense
c.697G>A	p.E233K	Missense
c.701A>G	p.H234R	Missense
c.702C>A	p.H234Q	Missense
c.703G>C	p.D235H	Missense
c.703G>T	p.D235Y	Missense
c.706G>T	p.G236C	Missense
c.749C>T	p.A250V	Missense
c.788C>G	p.S263C	Missense
c.788C>T	p.S263F	Missense
c.796A>T	p.S266C	Missense
c.803G>C	p.R268P	Missense
c.841T>A	p.C281S	Missense
c.911A>G	p.Y304C	Missense
c.914A>G	p.Y305C	Missense
c.932G>A	p.C311Y	Missense
c.934C>T	p.R312C	Missense
c.1169G>A	p.W390*	Nonsense
c.1177C>T	p.R393*	Nonsense
c.1285C>T	p.Q429*	Nonsense
c.130C>T	p.Q44*	Nonsense
c.1345C>T	p.Q449*	Nonsense
c.2203G>T	p.E735*	Nonsense
c.2434G>T	p.E812*	Nonsense
c.2728C>T	p.R910*	Nonsense
c.2785C>T	p.Q929*	Nonsense
c.3047G>A	p.W1016*	Nonsense
c.3100A>T	p.R1034*	Nonsense
c.3107C>A	p.S1036*	Nonsense
c.3242G>A	p.W1081*	Nonsense
c.3313C>T	p.Q1105*	Nonsense
c.3616C>T	p.R1206*	Nonsense
c.3735G>A	p.W1245*	Nonsense

c.3904C>T	p.Q1302*	Nonsense
c.470G>A	p.W157*	Nonsense
c.106-1G>C	NA	Splice site
c.1244+2T>G	NA	Splice site
c.1308+2_1308+5delTAGG	NA	Splice site
c.1308G>C	NA	Splice site
c.1309-1G>A	NA	Splice site
c.1584+5G>A	NA	Splice site
c.1585-1G>C	NA	Splice site
c.1786+1G>A	NA	Splice site
c.3044+1G>A	NA	Splice site
c.330+1G>A	NA	Splice site
c.331-1G>A	NA	Splice site
c.331-2_331-1delAG	NA	Splice site
c.414+1G>A	NA	Splice site
c.415-1G>A	NA	Splice site
c.686+1G>A	NA	Splice site
c.686+4T>G	NA	Splice site
c.687-2A>G	NA	Splice site
c.687-2delA	NA	Splice site
c.1007_1025dup19	p.D343Lfs*53	Frameshift
c.106_107delAG	p.S36Lfs*102	Frameshift
c.1095_1112del18	p.W365_R370del	Frameshift
c.1456_1457delAT	p.M486Vfs*47	Frameshift
c.1460delG	p.C487Sfs*10	Frameshift
c.1783_1784delTT	p.L595Gfs*19	Frameshift
c.1885delA	p.R629Efs*69	Frameshift
c.1922delA	p.E641Gfs*57	Frameshift
c.2000delA	p.N667Tfs*31	Frameshift
c.2042delA	p.K681Sfs*17	Frameshift
c.2173delC	p.Q725Kfs*53	Frameshift
c.2259delA	p.C754Afs*24	Frameshift
c.2279delG	p.G760Vfs*18	Frameshift
c.2348_2420+10del	p.A783Gfs*36	Frameshift
c.2376_2401del	p.A793Pfs*43	Frameshift
c.2455delG	p.A819Lfs*24	Frameshift
c.2549_2550delAT	p.D850Gfs*7	Frameshift
c.291_319del	p.E98Pfs*31	Frameshift
c.2939_2952del14	p.A980Gfs*3	Frameshift
c.3198_3199delCT	p.C1067Sfs*30	Frameshift
c.3220_3223delTACC	p.Y1074Afs*46	Frameshift
c.3254_3255delCT	p.S1085Cfs*12	Frameshift
c.334delG	p.A112Qfs*18	Frameshift
c.3543delG	p.Q1183Rfs*43	Frameshift
c.3587delA	p.H1196Pfs*30	Frameshift

c.3657_3658insT	p.P1220Sfs*12	Frameshift
c.372_373insGT	p.R125Vfs*6	Frameshift
c.3770dupT	p.L1258Vfs*36	Frameshift
c.3779dupT	p.M1260Ifs*34	Frameshift
c.4015delA	p.I1339Sfs*21	Frameshift
c.4034delA	p.N1345Tfs*15	Frameshift
c.4050delC	p.E1351Rfs*9	Frameshift
c.4091dupA	p.H1364Qfs*24	Frameshift
c.4119delG	p.Q1374Sfs*22	Frameshift
c.4143dupA	p.E1382Rfs*6	Frameshift
c.708delC	p.A237Rfs*12	Frameshift
c.709dupG	p.A237Gfs*153	Frameshift
c.717delC	p.S240Afs*9	Frameshift
c.724_729delTGCGGC	p.C242_G243del	Frameshift
c.762_774del13	p.P256Sfs*12	Frameshift
c.768_774delCCGCGCC	p.R257Afs*13	Frameshift
c.768_774dupCCGCGCC	p.G259Pfs*133	Frameshift
c.82dupT	p.W28Lfs*111	Frameshift
c.3844_3960del	p.T1282_T1320del	Inframe del
c.2931_2936delTGCCCG	p.C977_R979delinsW	Inframe indel
c.794_796delGCAinsCCT	p.C265_S266delinsSC	Inframe indel
c.964_970delTGCACCTinsGGCAGAC	p.C322_F324delinsGRL	Inframe indel
exon 15-16 deletion	NA	Copy number variation
exon 24-25 deletion	NA	Copy number variation
exon 27 deletion	NA	Copy number variation
exon 7-11 deletion	NA	Copy number variation
exon 7-8 deletion	NA	Copy number variation
